# Using Health Information Technology and Data to Improve Chronic Disease Outcomes in Federally Qualified Health Centers in Maryland

**DOI:** 10.5888/pcd13.160445

**Published:** 2016-12-29

**Authors:** Erica A. Smith, Judy Lapinski, Judy Lichty-Hess, Kristi Pier

**Affiliations:** 1 Maryland Department of Health and Mental Hygiene, Baltimore, Maryland; 2 Mid-Atlantic Association of Community Health Centers, Lanham, Maryland.

## Abstract

Federally Qualified Health Centers provide health care services to underserved communities and vulnerable populations. In Maryland, the burden of chronic disease is high among Federally Qualified Health Center patients. Electronic health records (EHRs) are becoming more widely used, and effective use of EHR data may improve chronic disease outcomes. This article describes the process of developing a data aggregation and analytics platform to support health centers in using population health data based on standardized clinical quality measures. This data warehouse, capable of aggregating EHR data across multiple health centers, provides opportunities for benchmarking and elicits a discussion of quality improvement, including identifying and sharing clinical best practices. Phase 1 of the project involved the strategic engagement of health center leadership and staff to get buy-in and to assess readiness. Phase 2 established the technological infrastructure and processes to support data warehouse implementation and began the process of information sharing and collaboration among 4 early adopters. Phase 3 will expand the project to additional health centers and continue quality improvement efforts. The health information technology marketplace is rapidly changing, and staying current will be a priority so that the data warehouse remains a useful quality improvement tool that continues to meet the demands of Maryland health centers. Ongoing efforts will also focus on ways to further add value to the system, such as incorporating new metrics to better inform health center decision making and allocation of resources. The data warehouse can inform and transform the quality of health care delivered to Maryland’s most vulnerable populations, and future research should focus on the ability of health centers to translate this potential into actual improvements.

## Introduction

Approximately 1 in 14 people in the United States access health care through a Federally Qualified Health Center (FQHC) (1). FQHCs provide primary and preventive health care services to vulnerable populations, including the medically underserved and uninsured. In addition to primary and preventive care, FQHCs provide services in women’s health, behavioral health, substance abuse, dental health, pharmacy, and social work and enabling services, among others. There are 17 FQHCs in Maryland that serve more than 300,000 patients. Most (91.0%) of these patients have a household income less than 200% of the federal poverty level (2). Almost half (49.9%) are eligible for Medicaid, and 18.7% are uninsured (Table 1).

**Table 1:** Demographic Characteristics and Chronic Disease Status of Patients in Maryland Federally Qualified Health Centers^a^

Characteristic	Percentage
**Patients**	
Household income <200% of the federal poverty level	91.0
Medicaid/CHIP recipients	49.9
Medicare	9.6
Third party insurance	21.8
Uninsured	18.7
Racial/ethnic minority	67.1
Homeless	5.0
**Chronic disease burden**	
Patients with hypertension	24.9
Patients with blood pressure control (patients with hypertension with blood pressure <140/90 mm Hg)	62.9
Patients with diabetes	11.6
Patients with uncontrolled diabetes (patients with diabetes with HbA1c >9%)	27.9

Abbreviations: CHIP, Children’s Health Insurance Program; HbA1c, hemoglobin A1c.

a Data obtained from the Health Resources and Services Administration, Maryland Uniform Data System, 2015. A total of 17 health centers served 303,352 patients (2).

The burden of chronic disease is high in the Maryland FQHC population. Approximately 1 in 4 patients has hypertension, and only 62.9% of patients with hypertension have their blood pressure under control (<140/90 mm Hg). Additionally, more than 1 in 9 patients has diabetes, which is poorly controlled (hemoglobin A1c [HbA1c] >9%) among more than one quarter (Table 1).

Some research suggests that electronic health record (EHR) use does not lead to improved medical care (3,4). However, these results were based on old data sets and different types of practices (ie, non-FQHC ambulatory care settings). Other studies found that increased health information technology capacity in FQHCs was associated with improved quality of care and that safety-net practices with EHRs demonstrate higher levels of diabetes care and better outcomes compared with FQHCs that use paper-based systems (5,6).

As EHR adoption continues to spread and health care systems address obstacles such as interoperability, health information technology can potentially transform health care delivery in the United States. Recognizing this, the federal government is committed not only to adopting EHR technology but also to using it meaningfully. Specifically, the 2009 Health Information Technology for Economic and Clinical Health Act authorized payments through Medicare and Medicaid to incentivize the meaningful use of EHRs to achieve specified improvements in health care delivery (7).

The Health Resources and Services Administration (HRSA) requires FQHCs to report EHR-generated data annually to the Uniform Data System (UDS) and evaluates the centers on standardized clinical quality measures that emphasize health outcomes and quality of care (8). Historically, HRSA has awarded additional quality incentives to FQHCs across the country that demonstrate significant improvement on key measures. Despite the availability of aggregated UDS data, the Mid-Atlantic Association of Community Health Centers (MACHC) and its member organizations recognized the need for a local data aggregation and analytics platform, a data warehouse, to support FQHCs in effectively using population health data to inform quality improvement efforts. This data aggregation and management strategy was identified as a priority goal by MACHC’s governing board to help drive reporting, analytics, business decision-making, and most importantly, clinical transformation across its member health centers. This article describes the process of developing and implementing a data warehouse to aggregate EHR data across multiple health centers for the purposes of benchmarking, identifying, and sharing clinical best practices and for establishing systems to improve chronic disease outcomes.

## Data Warehouse Development and Implementation

MACHC is a federally designated Primary Care Association, which provides training and technical assistance to all Maryland and Delaware FQHCs. MACHC was thus well positioned to coordinate and implement a large-scale data warehouse to improve population health management in all Maryland FQHCs. In 2014, with support from the Maryland Department of Health and Mental Hygiene and other key partners, MACHC began planning for a data warehouse to extract data from the FQHC EHRs, aggregate these data into a centralized system, and regularly report data on standardized measures back to FQHCs in a useable dashboard format.

The MACHC board of directors oversees the data warehouse project, and MACHC formed the Community Care Informatics Center (CCIC) to carry out the project’s scope of work. The project plan for fully developing the CCIC spans 4 years and is structured in 3 phases.

### Phase 1: Engage partners

During Phase 1, CCIC staff engaged FQHC leadership, quality improvement staff, and information technology staff to better understand existing data management structures, current successes, and challenges within the FQHCs as well as their development needs. The CCIC developed an assessment survey and met in person with each FQHC team for an information-gathering session that typically lasted approximately 2 hours. During these sessions, the teams and CCIC staff discussed factors such as the health centers’

- Strategic vision, goals, and plans with regard to using data to improve clinical outcomes.
- EHR use and workflow.
- Successes and areas for improvement in using data.
- Quality and consistency of clinical documentation in EHR.
- Existing tools for communicating data and outcomes, both internally and externally.
- Grant and program compliance, requirements, and challenges.

In Phase 1, the CCIC focused on the value added to FQHCs through provision of information, resources, and partnerships to improve health care quality and health outcomes. The CCIC also led a vendor analysis of available population health technologies to interface with the FQHCs’ EHRs, pull relevant data from the EHRs, and aggregate the data into another platform (ie, the data warehouse). After assessing various options, the CCIC subsequently identified 2 vendors as key strategic partners, since many FQHCs were already working with 1 of the 2 vendors. Both vendors were FQHC-focused and understood the nuances and special reporting requirements of health centers. The CCIC purchased the data warehouse software that analyzes and aggregates the EHR information.

The CCIC determined that data ownership would reside with the individual FQHCs and that decisions on data sharing and other procedures would be made collectively by the health centers. The CCIC established an advisory council, comprising clinical and quality improvement staff from all Maryland FQHCs, to develop procedures that guide the data warehouse implementation. The CCIC later formed a contributors committee, whose membership includes the CEOs of the early adopter health centers. The contributors committee provides strategic oversight and has decision-making authority about new requests for data and the sharing of CCIC-specific data with community partners and other stakeholders.

Contributing health centers entered into a participation agreement with MACHC as well as a user agreement with MACHC’s selected software vendors. The participation agreement allowed the CCIC to aggregate and share data with other participating health centers and stakeholders as agreed on by the contributors committee. It also detailed the roles and responsibilities of MACHC staff regarding overall execution of the project from an information technology and management perspective, technical support to health centers during implementation and data validation, protection of data, and reporting expectations. Roles and responsibilities of the FQHCs included providing adequate staff resources to work with the CCIC staff during implementation and data validation, committing to implement 1 of 2 predetermined population health software systems to allow for data aggregation, and ensuring availability of data.

### Phase 2: Building and validating

In Phase 2, the CCIC advisory committee made operational decisions, including selecting specific clinical quality measures to be reported and approving the data validation process. The final agreed-on clinical dashboard for the data warehouse included measures that the advisory council believed were the most critical to focus on during the first years of the project. The council strove to include measures that aligned with federal, state, and grant-related reporting standards such as UDS, Meaningful Use, Healthcare Effectiveness Data and Information Set (HEDIS), and National Quality Forum (NQF) (9–12). The CCIC developed a crosswalk to indicate each measure’s overlap in these commonly used, national standards.

Reporting accurate, consistent data is critical to the integrity of the project and its usefulness to the FQHCs. Data validation can be complicated and tedious as a result of varying workflows and documentation practices across health centers. Defining a clear process for initial and ongoing validation was essential. CCIC staff worked directly with the FQHCs to establish the reliability and validity of data in their EHR systems and provided training as needed. Ultimately, FQHCs signed off that the information in the CCIC data warehouse mirrored the data in the center’s EHR and encompassed all data from the EHR necessary to accurately report each metric. The advisory council vetted and agreed on this data validation process, which was developed by the CCIC.

In collaboration with the EHR vendors and the 2 population health software vendors, the CCIC provided technical expertise when needed in the implementation of the population health systems that health centers purchased. CCIC staff collaborated with the FQHCs on implementation alignment aspects to ensure standardization of population health software setup and utilization functionality. Although there is functionality with these software products, the health centers also contributed significantly to necessary customizations in many cases to better manage population health efforts at the health center level. The CCIC also provided as-needed technical guidance, such as assisting FQHCs with systems assessments and discussing data readiness.

As of May 2016, 4 FQHCs were reporting to the data warehouse. The first dashboard had 29 measures and included specifically defined numerators and denominators. These data will serve as a baseline for future quality improvement work. The data warehouse gives FQHCs the capability to access aggregated health center data more frequently than once per year, as required for UDS, through quarterly dashboard reports provided by the CCIC. Health centers can also use their population health programs to review individual reports and to evaluate their individual progress at any time.

Within the first year of reporting, the delivery of preventive services and clinical outcomes varied widely among the 4 contributing FQHCs. The first dashboard report pulled EHR data from the 4 health centers for April 1, 2015, through March 31, 2016. During that time, the percentage of patients with hypertension whose blood pressure was adequately controlled during the measurement year ranged from 49.6% to 73.7%. During this same period, the percentage of patients aged 18 to 75 years with diabetes (type 1 or type 2) whose most recent HbA1c level during the measurement year was greater than 9.0% (ie, demonstrating poor blood glucose control) or whose data were missing ranged from 33.3% to 70.0%. Additional dashboard results are described in Table 2.

**Table 2:** Hypertension and Diabetes Screening and Control Measures From 4 Federally Qualified Health Centers (FQHCs) in Maryland, 2015–2016

Measure	Aggregate Percentage of Patients in Contributing FQHCs
**Hypertension**	
Blood pressure screening: percentage of patients aged 18 years or older who are screened for high blood pressure (Physician Quality Reporting System measure 317).	82.2
Blood pressure control: percentage of patients aged 18–85 years who had a diagnosis of hypertension and whose blood pressure was adequately controlled during the measurement year (National Quality Forum measure 18).	62.6
**Diabetes**	
Diabetic eye examination: the percentage of patients aged 18–75 years with diabetes (type 1 or type 2) who had a retinal or dilated eye exam or a negative retinal exam (no evidence of retinopathy) by an eye care professional (National Quality Forum measure 55).	7.0
Diabetic foot examination: the percentage of patients aged 18–75 years with diabetes (type 1 or type 2) who received a foot examination (visual inspection and sensory examination with monofilament and a pulse exam) during the measurement year (National Quality Forum measure 56).	45.9
Diabetic A1c testing: the percentage of patients aged 18–75 years with diabetes (type 1 or type 2) who received an HbA1c test during the measurement year (National Quality Forum measure 57).	68.4
HbA1c poor control: The percentage of patients aged 18–75 years with diabetes (type 1 or type 2) whose most recent HbA1c level during the measurement year was greater than 9.0% (poor control) or was missing a result, or if an HbA1c test was not done during the measurement year (National Quality Forum Measure 59).	49.2

Abbreviation: HbA1c, hemoglobin A1c.

To facilitate effective use of the aggregate data for clinical and quality improvement, MACHC formed a contributors’ workgroup, consisting of clinical and quality staff from the contributing health centers. The contributors’ workgroup meets at least 6 times per year and focuses on developing and sharing evidence-based and innovative best practices as well as on prioritizing efforts to improve population health outcomes. As more centers are able to successfully report data into the CCIC data warehouse, participation in the contributors’ workgroup will grow.

The difference in FQHCs’ ability to demonstrate success on key performance indicators suggests there is an opportunity for peer-to-peer learning. In June 2016, the contributors’ workgroup identified 3 initial priority focus areas: hypertension control, diabetes control, and colorectal cancer screening. MACHC plays a facilitation role in the workgroup, encouraging discussion on quality improvement while FQHCs that have demonstrated success in each priority area lead workgroup discussions on that topic.

### Phase 3: Replication and continuous quality improvement

During Phase 3, MACHC will use the infrastructure to replicate the process to include more FQHCs in the data warehouse. This final phase will also include continual quality improvement efforts, using data to inform health systems transformation efforts. Although new challenges to bringing on additional health centers will occur largely because of the varying EHR and population health information technology software, MACHC will leverage lessons learned and standardized processes, such as data validation and dashboard development processes, to ease onboarding for newly joining health centers.

Initially, only predetermined clinical quality data were included in the dashboard, with the sole focus being clinical quality improvement. Evaluating clinical metrics in the aggregate and individually, as well as comparing them with state and federal values when available, will allow the CCIC to continue to pinpoint best clinical practices and leverage knowledge and expertise from health centers that are performing well in certain areas. The CCIC will also continue to partner with organizations such as the American Cancer Society and Kaiser Permanente to provide evidence-based protocols and additional training for health centers to support improvement efforts. Although Maryland health centers are performing above the national average in many measures, these combined efforts will allow for focused continual quality improvement leading to even better health outcomes for some of the most vulnerable populations in the state.

Plans include adding data on social determinants of health and financial information to the clinical measures in the dashboard. Patients of FQHCs often have many comorbidities in addition to social and economic challenges. Managing these barriers alongside patients is a critical step in improving health outcomes. Including social barriers and cost-of-care information will allow health centers to better allocate resources for care of patients with these complex issues. Obtaining cost-of-care information will probably require an additional interface with third-party payers, but the data warehouse may be able to glean this information from the practice management portion of EHRs, which manage billing and collections.

## Challenges

As with most projects, the CCIC learned to adapt, because technology is ever-changing and the health care landscape continues to evolve. One initial challenge the CCIC faced was managing the work through 2 population health platforms. To address this challenge, the CCIC staff initially focused on FQHCs using the same population health platform software to become early adopters. Onboarding additional health centers, using either of the 2 selected platforms, will continue throughout Phase 3 of the project. MACHC will also continue to assess new technologies and monitor changes in the health information technology marketplace to ensure that the data warehouse remains a useful and relevant tool.

Overall, cost was a major challenge for both the health centers and MACHC. Although MACHC received significant grant funds from the State of Maryland — made possible by the Centers for Disease Control and Prevention’s (CDC’s) support to all 50 states and the District of Columbia via the State Public Health Actions to Prevent and Control Diabetes, Heart Disease, Obesity and Associated Risk Factors and Promote School Health (State Public Health Actions) program — these funds were still below the actual costs of the project. At the local level, the cost of the population health software products was prohibitive for some health centers. Recently, HRSA announced the availability of information technology grant funds, which many FQHCs chose to use to purchase the necessary population health software product. MACHC continues to work to leverage other available resources to continue the project and ensure its sustainability.

## Conclusion

The innovative data warehouse project in Maryland can inform and transform the quality of health care delivered to the state’s most vulnerable populations. However, the project is still in its early stages and has yet to translate this tremendous potential into real-world improvements. Future research should revisit the data warehouse efforts to further evaluate its reach and impact. Future research should focus not only on progress on clinical outcomes and the delivery of preventive services but also on changes in clinical practice resulting from data sharing, benchmarking, and collaboration around quality improvement.
